# RACIAL AND ETHNIC DIFFERENCES IN LUNG CANCER SCREENING ELIGIBILITY AND HEALTH CARE UTILIZATION AMONG OLDER SMOKERS

**DOI:** 10.1093/geroni/igac059.2836

**Published:** 2022-12-20

**Authors:** Jaqueline Avila, Ugoji Nwanaji-Enwerem, Randi Williams, Hilary Robbins, Jasjit Ahluwalia

**Affiliations:** Brown University School of Public Health, Providence, Rhode Island, United States; Warren Alpert School of Medicine, Brown University, Providence, Rhode Island, United States; Lombardi Comprehensive Cancer Center, Georgetown University Medical Center, Washington, District of Columbia, United States; International Agency for Research on Cancer, Lyon, Rhone-Alpes, France; Brown University School of Public Health, Providence, Rhode Island, United States

## Abstract

The United States Preventive Task Force (USPSTF) expanded the lung cancer screening guidelines with low-dose CT scan (LDCT) to narrow racial and ethnic screening disparities. However, there is a need to examine if eligible individuals can access and use health care. The objective was to examine racial and ethnic differences in LDCT eligibility and health services access and utilization among LDCT-eligible individuals. Data comes from adults 50 to 80 years old in the 2018 Health and Retirement Study (HRS), with at least a 20 pack-year smoking history who currently smoke, or have quit < 15 years ago (n=7,624). The outcomes were LDCT eligibility, access to health care (health insurance and usual place of care), and health care use (visits to the doctor). White individuals were more likely to be LDCT eligible than Black and Hispanic individuals. Among those LDCT eligible, Hispanic individuals were less likely to have insurance (OR:0.43, [95%CI: 0.21; 0.86]) and to visit the doctor than White individuals (OR: 0.38 [0.19; 0.76]). Compared to White individuals, Black individuals were more likely to say their usual place of care was the ER or “other” place (OR: 2.65 [1.63; 3.32] and Hispanic individuals were more likely to say they do not have a usual place of care (OR: 1.94 [1.10; 3.41]). Expanding the criteria for lung cancer screening may not be enough to reduce racial and ethnic disparities. More efforts should address racial and ethnic disparities in the implementation of lung cancer screening, including access and use of health care.

